# Supplementary material for: Genome-Wide Identification and Transcriptomic Analysis of MicroRNAs Across Various Amphioxus Organs Using Deep Sequencing
Source: Front Genet. 2019 Sep 26;10:877. doi: 10.3389/fgene.2019.00877 (PMC6775235; doi:10.3389/fgene.2019.00877)
Supplement: Supplementary File 1 — Taqman probe information of randomly selected miRNAs used in qRT-PCR analysis. [file Presentation_1.zip › Supplementary File 7.pdf]

| | | |
|---|---|---|
| Provisional ID | : | scaffold65_2653 |
| Score total | : | 12032582.3 |
| Score for star read(s) | : | 3.9 |
| Score for read counts | : | 12032573.5 |
| Score for mfe | : | 1.9 |
| Score for randfold | : | |
| Score for cons. seed | : | 3 |
| Total read count | : | 23601386 |
| Mature read count | : | 23601324 |
| Loop read count | : | 0 |
| Star read count | : | 62 |

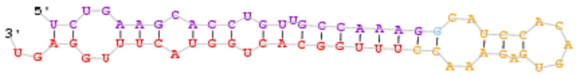

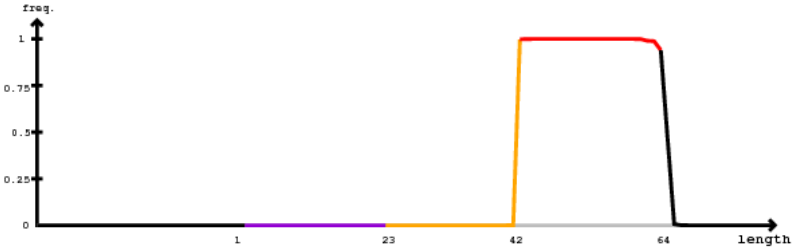


**Star** **Mature**

```
5'- ugaagucuuaagugauccaccugcuugauucugaagcaccuguugccaaaggcauccacagugagaaaccuuuggcacugguacuuuggagucaagauggugacaaua  -3'   obs
    ugaagucuuaagugauccaccugcuugauucugaagcaccuguugccaaaggcauccacagugagaaaccuuuggcacugguacuuuggagucaagauggugacaaua         exp
    ....(((......))).((((..((((((((..(((.(((.((.((((((((..(((.....).))..))))))))).))).)))..)))))))).))))......   reads   mm   sample
    .............................ucugaagcaccuguuAccaaag.........................................................   38   1   seq
    .............................ucugaagcaccuguuAccaaagg........................................................   24   1   seq
    ....................................................................Ucuuuggcacugguacuuuggagu................   74   1   seq
    .....................................................................cuuuggcacugguacuuu.....................   10   0   seq
    .....................................................................cuuuggcacugguacuuugg...................   924   0   seq
    .....................................................................cuuuggcacugguacuuugA...................   24   1   seq
    .....................................................................cuuuggcacugguacuuugga..................   24   0   seq
    .....................................................................cuuuggcacugguacuuuggag.................   486   0   seq
    .....................................................................cuuuggcacugguacuuuggaA.................   13   1   seq
    .....................................................................cuuuggcacugguacuuuggagA................   283   1   seq
    .....................................................................cuuuggcacuggCacuuuggagu................   10   1   seq
    .....................................................................cuuuggcacugguacuuuggGgu................   36   1   seq
    .....................................................................Uuuuggcacugguacuuuggagu................   163   1   seq
    .....................................................................cuuugAcacugguacuuuggagu................   12   1   seq
    .....................................................................cuuuggcacuAguacuuuggagu................   13   1   seq
    .....................................................................cuuuggcacugguacuuuggagG................   182   1   seq
    .....................................................................cuuuggcGcugguacuuuggagu................   14   1   seq
    .....................................................................cuuuggcacugguacuuuggagu................   9414   0   seq
    .....................................................................cuuuggcacCgguacuuuggagu................   25   1   seq
    .....................................................................cuuCggcacugguacuuuggagu................   27   1   seq
    .....................................................................cuCuggcacugguacuuuggagu................   21   1   seq
    .....................................................................cuuuggcacugguacuuuggagC................   656   1   seq
    .....................................................................cuuuggcacugguacuCuggagu................   12   1   seq
    .....................................................................cuuuggcacugguacuuCggagu................   24   1   seq
    .....................................................................cuuuggcacugguacCuuggagu................   15   1   seq
    .....................................................................cuuuggcacugguacuuuggaguA...............   81   1   seq
    .....................................................................cuuuggcacugguacuuuggaguU...............   40   1   seq
    .....................................................................cuuuggcacugguacuuuggaguAa..............   64   1   seq
    ......................................................................uuuggcacugguacuuug....................   16104   0   seq
    ......................................................................uuuggcacugguGcuuug....................   10   1   seq
    ......................................................................Auuggcacugguacuuug....................   10   1   seq
    ......................................................................uuuggcacCgguacuuug....................   34   1   seq
    ......................................................................uuuggcacugguacCuug....................   46   1   seq
```

novel_mir1

| | | |
|---|---|---|
| Provisional ID | : | scaffold8_32724 |
| Score total | : | 5059.8 |
| Score for star read(s) | : | 3.9 |
| Score for read counts | : | 5054.5 |
| Score for mfe | : | 2 |
| Score for randfold | : | |
| Score for cons. seed | : | -0.6 |
| Total read count | : | 9926 |
| Mature read count | : | 9863 |
| Loop read count | : | 0 |
| Star read count | : | 63 |

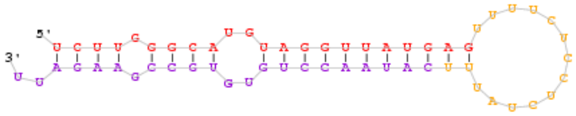

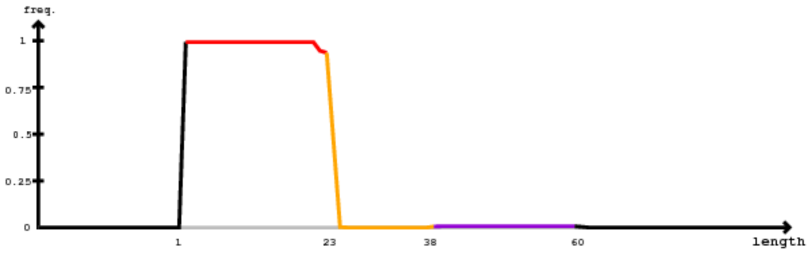


```
                    Mature                      Star
5'- uuccguugcucucuuggaggucuugggcauguagguuaugaguuucuccucuauuucauaaccugugugccgaagauuuucaauggaacuuuugccagugagcgugaag  -3'  obs
    uuccguugcucucuuggaggucuugggcauguagguuaugaguuucuccucuauuucauaaccugugugccgaagauuuucaauggaacuuuugccagugagcgugaag       exp
    (((...(((((...((((((((((.((((..(((((((((((............))))))))))..)))).))))))))))).(((........)))..))))).))).   reads  mm  sample
    ....................ucuugggcauguagguuaug.....................................................................   469    0   seq
    ....................ucuugggcauguagguuauga....................................................................   101    0   seq
    ....................ucuugggcGuguagguuaugag...................................................................   13     1   seq
    ....................ucuugggAauguagguuaugag...................................................................   11     1   seq
    ....................Ccuugggcauguagguuaugag...................................................................   13     1   seq
    ....................ucuugggcauguGgguuaugag...................................................................   17     1   seq
    ....................ucuugggcauguagguuaugGg...................................................................   15     1   seq
    ....................ucuugggcauguagguuaugag...................................................................   3943   0   seq
    ....................ucuugggcauguagguuaugaA...................................................................   76     1   seq
    ....................ucCugggcauguagguuaugag...................................................................   11     1   seq
    ....................ucuugggcauguagguuaugagu..................................................................   1604   0   seq
    ....................ucuugggcauguagguuaugagC..................................................................   104    1   seq
    ....................ucuugggcauguagguuaugagA..................................................................   3365   1   seq
    ....................ucuugggcauguagguuaugaCu..................................................................   13     1   seq
    ....................ucuugggcauguagguuaugGgu..................................................................   10     1   seq
    ....................Ccuugggcauguagguuaugagu..................................................................   14     1   seq
    ....................ucuugggcauguagguuaugagG..................................................................   66     1   seq
    ....................ucuugggcauguagguuaugaguu.................................................................   18     0   seq
    ........................................................cauaaccugugugccgaagauu...............................   48     0   seq
    .........................................................auaaccugugugccgaagauu...............................   15     0   seq
```

novel_mir2

| | | |
|---|---|---|
| Provisional ID | : | scaffold5_28188 |
| Score total | : | 0.8 |
| Score for star read(s) | : | -1.3 |
| Score for read counts | : | 0 |
| Score for mfe | : | 2.7 |
| Score for randfold | : | |
| Score for cons. seed | : | -0.6 |
| Total read count | : | 516 |
| Mature read count | : | 516 |
| Loop read count | : | 0 |
| Star read count | : | 0 |

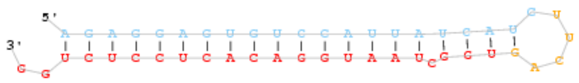


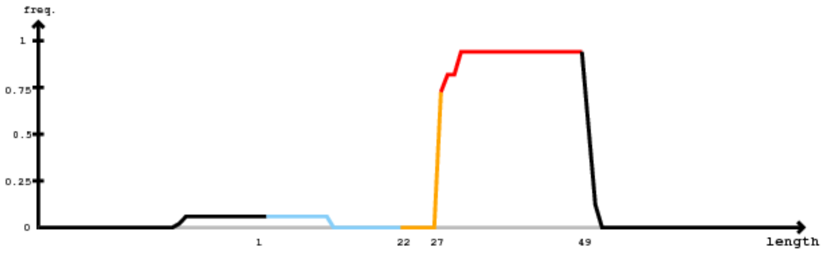


```
                          Star                  Mature
5'- aucaaagggucgcaaggcaguauucauuggcuagaggaguguccauuaucaucuucaguggcuaauggacacuccucuggccauuguggauacugccucuccaccguaugau  -3'   exp
    ((((.(.(((.(..((((((((((((((((((((((((((((((((((((((.....)))).)))))))))))))))))))))..)))))))))))))..).))).).))))   reads   mm   sample
    ...................guauucauuggcuagaggagugu......................................................................   11      0    seq
    ....................uauucauuggcuagaggagugu......................................................................   21      0    seq
    ..........................................................uggcuaauggacacuccucugg................................   371     0    seq
    ..........................................................uggcuaauggacacuccucugU................................   27      1    seq
    ...........................................................ggcuaauggacacuccucugg................................   51      0    seq
    .............................................................cuaauggacacuccucuggcc..............................   67      0    seq
```

novel_mir3

| | | |
|---|---|---|
| Provisional ID | : | scaffold6_3322 |
| Score total | : | 0.6 |
| Score for star read(s) | : | -1.3 |
| Score for read counts | : | 0 |
| Score for mfe | : | 2.5 |
| Score for randfold | : | |
| Score for cons. seed | : | -0.6 |
| Total read count | : | 365 |
| Mature read count | : | 365 |
| Loop read count | : | 0 |
| Star read count | : | 0 |

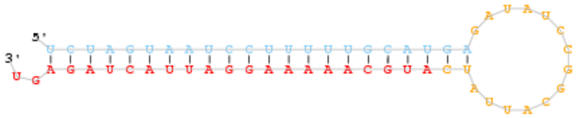


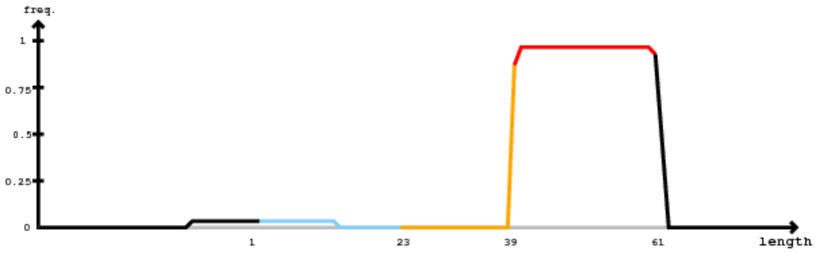


Star Mature

```
5'- uagagccagacagcaucuagccguggacgacucuaguaauccuuuuugcaugagauauccggcauuaucaugcaaaaaggauuacuagagucauccacagcuagacagcau  -3'   exp
    ...........((.((((((.(((((.((((((((((((((((((((((((.............)))))))))))))))))))))))).))))).))))))..))..   reads   mm   sample
    .....................cguggacgacucuaguaauccu.....................................................................   13      0    seq
    .....................................................................augcaaaaaggauuacuagag.....................   14      0    seq
    .....................................................................augcaaaaaggauuacuagagu....................   278     0    seq
    .....................................................................augcaaaaaggauuacuagagA....................   20      1    seq
    .....................................................................augcaaaaaggauuacuagagC....................   17      1    seq
    ......................................................................ugcaaaaaggauuacuagagu....................   36      0    seq
```

novel_mir4

| | | |
|---|---|---|
| Provisional ID | : | scaffold81_31086 |
| Score total | : | 0.6 |
| Score for star read(s) | : | -1.3 |
| Score for read counts | : | 0 |
| Score for mfe | : | 2.5 |
| Score for randfold | : | |
| Score for cons. seed | : | -0.6 |
| Total read count | : | 154 |
| Mature read count | : | 154 |
| Loop read count | : | 0 |
| Star read count | : | 0 |

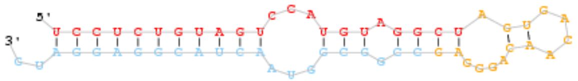


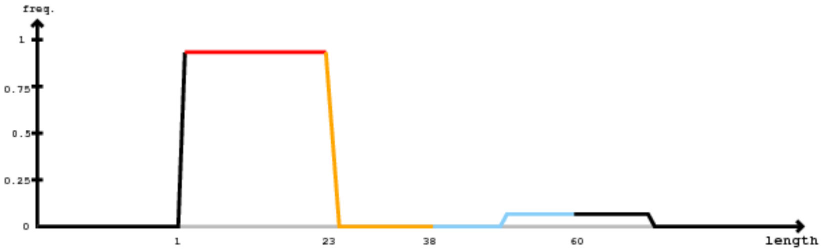


```
                   Mature                           Star
5'- aggcucaggccugggugccauccucuguaguccauguaggcuagugacaacagggagccggcgguaacuacggaggauggcacccaggcuaccgaagcuccacgggacucaa   -3'    exp
    .......((((((((((((((((((((((((...(((.((((.((....))....)))).)))...))))))))))))))))))))))))))).(((........))).......    reads     mm      sample
    ....................uccucuguaguccauguaggcu......................................................................    154       0       seq
    ..................................................................uacggaggauggUacccaggcu......................    11        1       seq
```

novel_mir5

| | | |
|---|---|---|
| Provisional ID | : | scaffold17_37153 |
| Score total | : | 2924.8 |
| Score for star read(s) | : | 3.9 |
| Score for read counts | : | 2919.4 |
| Score for mfe | : | 2.1 |
| Score for randfold | : | |
| Score for cons. seed | : | -0.6 |
| Total read count | : | 5738 |
| Mature read count | : | 5676 |
| Loop read count | : | 0 |
| Star read count | : | 62 |

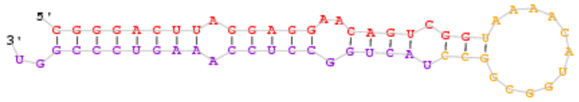


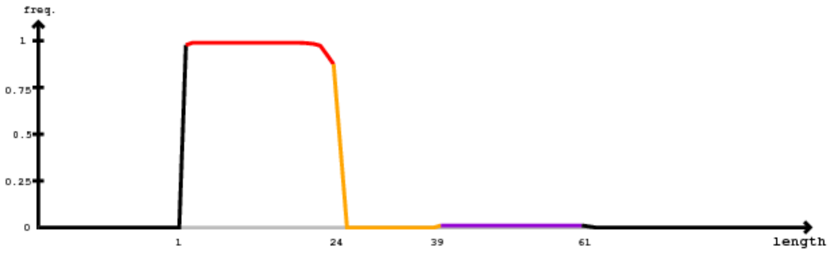


Mature Star

```
5'- ugagacuacgugugucegaucgggacuuaggaggaacagucgguaaaacauggcggccuacuggccuccaaagucccgguuggcauccguaaauuuuccucgguaaaauugacg -3'   obs
    ugagacuacgugugucegaucgggacuuaggaggaacagucgguaaaacauggcggccuacuggccuccaaagucccgguuggcauccguaaauuuuccucgguaaaauugacg        exp
    .((((.((((..((.(((((((((((((.(((((..((((.(((...........))).)))).))))).)))))))))))))))..))))...))))................   reads   mm   sample
    ....................cgggacuuaggaggaaca............................................................................   11      0    seq
    ....................cgggacuuaggaggaacag...........................................................................   21      0    seq
    ....................cgggacuuaggaggaacagu..........................................................................   54      0    seq
    ....................cgggacuuaggaggaacaguc.........................................................................   120     0    seq
    ....................cgggacuuaggaggaacaguA.........................................................................   29      1    seq
    ....................cgggacuuGggaggaacaguc.........................................................................   128     1    seq
    ....................cgggacuuGggaggaacagucg........................................................................   91      1    seq
    ....................cgggacuuaggaggaacagucg........................................................................   167     0    seq
    ....................cgggacuuaggaggaacaguAg........................................................................   26      1    seq
    ....................cgggacuuaggaggaacagucA........................................................................   11      1    seq
    ....................cgggacuuaggaggaacagucgA.......................................................................   57      1    seq
    ....................cgggacuuGggaggaacagucgg.......................................................................   1469    1    seq
    ....................cgggacuuaggaggaacagucgg.......................................................................   2497    0    seq
    ....................cgggacuuaggaggaacagucgC.......................................................................   17      1    seq
    ....................cgggacuuaggaggaacaguAgg.......................................................................   612     1    seq
    ....................cgggacuuaggaggGacagucgg.......................................................................   12      1    seq
    ....................cgggacuuaggaggaCcagucgg.......................................................................   218     1    seq
    ....................cgggacuuGggaggaacagucggu......................................................................   23      1    seq
    ....................cgggacuuaggaggaacagucggu......................................................................   25      0    seq
    ....................cgggacuuaggaggaacaguAggu......................................................................   21      1    seq
    .....................gggacuuaggaggaacaguAgg.......................................................................   10      1    seq
    .....................gggacuuaggaggaacaguAggu......................................................................   57      1    seq
    ..........................................................uacuggccuccaaagucccggu..................................   17      0    seq
    ..........................................................uacuggccuccaaagucccgAu..................................   45      1    seq
```

novel_mir6

| | | |
|---|---|---|
| Provisional ID | : | scaffold33_42244 |
| Score total | : | 2770.7 |
| Score for star read(s) | : | 3.9 |
| Score for read counts | : | 2764.4 |
| Score for mfe | : | 3 |
| Score for randfold | : | |
| Score for cons. seed | : | -0.6 |
| Total read count | : | 5434 |
| Mature read count | : | 5066 |
| Loop read count | : | 0 |
| Star read count | : | 368 |

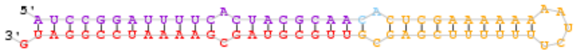


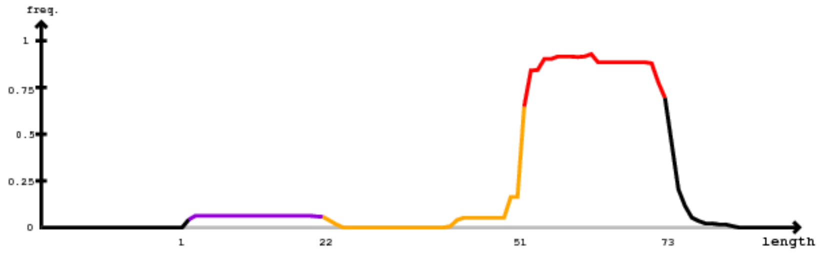


**Star** **Mature**

```
5'- ggcagaguagccuggguaccauccggauuuucacuacgcaacacucgaaaaaaaaucuuuuuuucgagcguugcguagcgaaaauccggaugguacccaggguaccggcag  -3'   obs
    ggcagaguagccuggguaccauccggauuuucacuacgcaacacucgaaaaaaaaucuuuuuuucgagcguugcguagcgaaaauccggaugguacccaggguaccggcag        exp
    .((.(.(((.(((((((((((((((((((((((.(((((((((.((((((((((.....)))))))))).))))))))).)))))))))))))))))))))).)))).))..  reads   mm   sample
    ....................auccggauuuucacuacgc........................................................................  21      0    seq
    ....................auccggauuuucacuacgca.......................................................................  12      0    seq
    ....................auccggauuuucacuacgcaa......................................................................  88      0    seq
    ....................Cuccggauuuucacuacgcaa......................................................................  21      1    seq
    ....................auccggauCuucacuacgcaa......................................................................  19      1    seq
    ....................auccggauuuucacuacgcaaU.....................................................................  87      1    seq
    .....................uccggauuuucacuacgcaa......................................................................  25      0    seq
    .....................uccggauuuucGcuacgcaaca....................................................................  95      1    seq
    ...........................................................uuuuucgagcguugcguagcg...............................  10      0    seq
    ...........................................................uuuuucgagcguugcguagcga..............................  28      0    seq
    ............................................................uuuucgagcguugcguag.................................  13      0    seq
    ............................................................uuuucgagcguugcguagcg...............................  27      0    seq
    ............................................................uuuucgagcguugcguagcAa..............................  14      1    seq
    ............................................................uuuucgagcguugcguagcga..............................  144     0    seq
    .............................................................uuucgagcguugcguagcAa..............................  18      1    seq
    .............................................................uuucgagcguugcguagcga..............................  53      0    seq
    ....................................................................cguugcguagcCaaaauccgg.....................  32      1    seq
    ....................................................................cguugcguagcCaaaauccgga....................  560     1    seq
    ....................................................................cguugcguagcgaaaauccgga....................  44      0    seq
    ....................................................................cguugcguagcCaaaauccggau...................  17      1    seq
    ......................................................................uugcguagcgaaaauccggau....................  248     0    seq
    ......................................................................uugcguagcgaaaauccggaug...................  859     0    seq
    ......................................................................uugcguagcgaaaaucUggaug...................  109     1    seq
    ......................................................................uugcgCagcgaaaauccggaug...................  15      1    seq
    ......................................................................uugcguagcAaaaauccggaug...................  102     1    seq
    ......................................................................uugcguagcgaaaauccggauU...................  410     1    seq
    ......................................................................uugUguagcgaaaauccggaug...................  17      1    seq
    ......................................................................uugcUuagcgaaaauccggaug...................  32      1    seq
    ......................................................................uugcguGgcgaaaauccggaug...................  13      1    seq
    ......................................................................uugcguagcgaaaGuccggaug...................  16      1    seq
    ......................................................................uugcguagcgaaaauccggauA...................  53      1    seq
    ......................................................................uugcguagcgaaaauccggaugU..................  462     1    seq
    ......................................................................uugcguagcgaaaauccggaugA..................  366     1    seq
```

novel_mir7

| | | |
|---|---|---|
| Provisional ID | : | scaffold110_23657 |
| Score total | : | 0.3 |
| Score for star read(s) | : | -1.3 |
| Score for read counts | : | 0 |
| Score for mfe | : | 2.2 |
| Score for randfold | : | |
| Score for cons. seed | : | -0.6 |
| Total read count | : | 149 |
| Mature read count | : | 149 |
| Loop read count | : | 0 |
| Star read count | : | 0 |

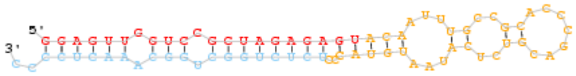

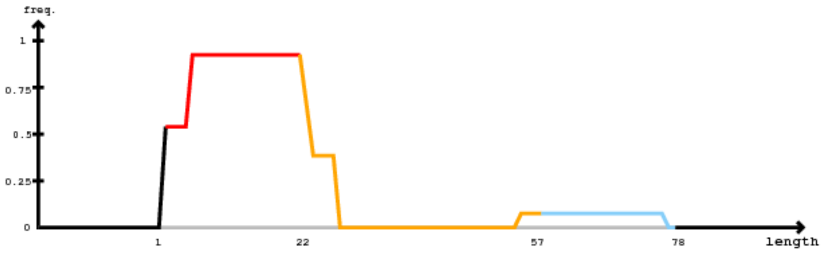


Mature Star

```
5'- agagauaacaugccaggggaguugguccgcuagagaguacaauuugccgcacccgacgucucauaaauguacccucucuggcuggcaaacuccccugguaauuuauuuuggcc  -3'  exp
    .(((((((..(((((((((((((.(((.(((((((((((((...((..((.......))..))...)))))..))))))))).))).)))))))))))))..)))))))....   reads  mm  sample
    .................Ugaguugguccgcuagagagu...........................................................................   55     1   seq
    .................gUaguugguccgcuagagagu...........................................................................   21     1   seq
    .................Agaguugguccgcuagagagua..........................................................................   11     1   seq
    .....................uugguccgcuagagaguacaGu......................................................................   32     1   seq
    .....................uugguccgcuagagaguacaUu......................................................................   30     1   seq
    .......................................................................cccucucuggcuggcaaacucc....................   12     0   seq
```

novel_mir8

| | | |
|---|---|---|
| Provisional ID | : | scaffold373_29810 |
| Score total | : | 0.3 |
| Score for star read(s) | : | -1.3 |
| Score for read counts | : | 0 |
| Score for mfe | : | 2.2 |
| Score for randfold | : | |
| Score for cons. seed | : | -0.6 |
| Total read count | : | 167 |
| Mature read count | : | 167 |
| Loop read count | : | 0 |
| Star read count | : | 0 |

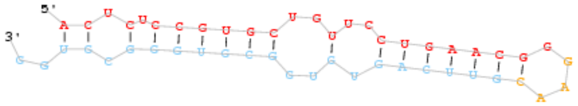


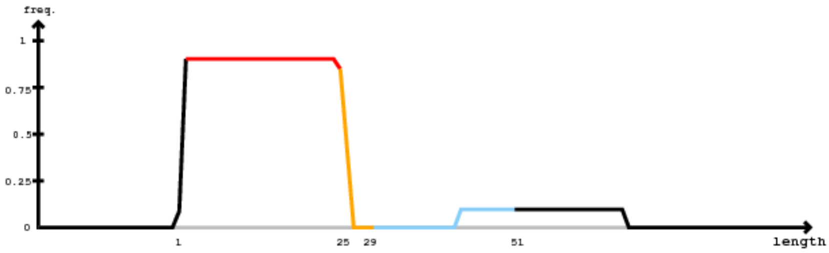


**Mature** **Star**

```
5'- augaguauggcggacuuccuacucuccgugcuguuccugaacgggaacguucaguguggcgugggcguggaccuggccugcaggcacccgguggcggaccuggccugcaggca  -3'  exp
    ..........((...(((.((.(.((((((..(..(((((((....))))))).)..))))))).)))))))..((((((((((..(((....))).....)))))))))).  reads  mm  sample
    ...................uacucuccgugcuguuccugaacgg.....................................................................  16     0   seq
    ....................acucuccgugcuguuccugaacg......................................................................  10     0   seq
    ....................acucuccgugcuguuccugaacgg.....................................................................  124    0   seq
    ....................acucuccgugcuguuccugaacggg....................................................................  17     0   seq
    .............................................................ugggcguggaccuggcAugcaggca...........................  18     1   seq
```

novel_mir9

| | | |
|---|---|---|
| Provisional ID | : | scaffold42_15258 |
| Score total | : | 0.2 |
| Score for star read(s) | : | -1.3 |
| Score for read counts | : | 0 |
| Score for mfe | : | 2.1 |
| Score for randfold | : | |
| Score for cons. seed | : | -0.6 |
| Total read count | : | 1199 |
| Mature read count | : | 990 |
| Loop read count | : | 0 |
| Star read count | : | 209 |

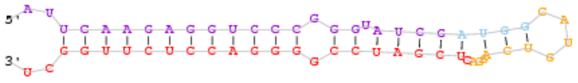

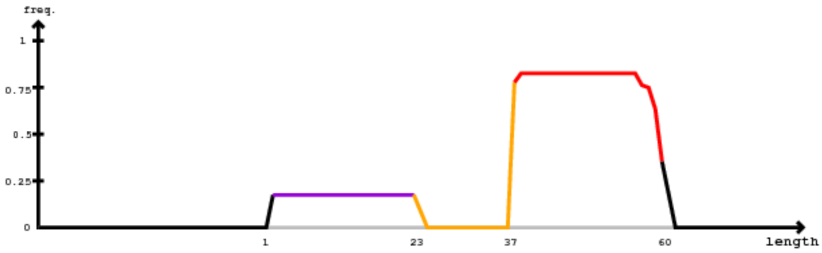


```
                                 Star                                       Mature
5'- caaucuuccagccgucauuucguacuuacucggauucaagagguccggguaucgaugg caugucaggacucgauccggggaccucuuggcuccgagucaagcacucugucg  -3'  obs
    caaucuuccagccgucauuucguacuuacucggauucaagagguccggguaucgauggcaugucaggacucgauccggggaccucuuggcuccgagucaagcacucugucg        exp
    ............((.((....((.((((((((((..(((((((((((.((.((((((((....)))....))))))).)))))))))))..))))))).))).)).)).))   reads   nm   sample
    .................................auucaagagguccggguaucg.........................................................   133     0    seq
    .................................auucaagagguccggguaucga........................................................   76      0    seq
    ....................................................................cucgauccggggaccucuu........................   76      0    seq
    ....................................................................cucgauccggggaccucuug.......................   16      0    seq
    ....................................................................cucgauccggggaccucuugg......................   137     0    seq
    ....................................................................cucgauccggggaccucuuggc.....................   258     0    seq
    ....................................................................cucgauccggggaccucuuggU.....................   70      1    seq
    ....................................................................cucgauccggggaccucuuggcu....................   23      0    seq
    ....................................................................cucgauccggggaccucuuggcA....................   306     1    seq
    ....................................................................cucgauccggggaccucuuggcG....................   46      1    seq
    .....................................................................ucgauccggggaccucuuggU.....................   10      1    seq
    .....................................................................ucgauccggggaccucuuggcu....................   48      0    seq
```

novel_mir10

| | | |
|---|---|---|
| Provisional ID | : | scaffold151_19686 |
| Score total | : | 0.1 |
| Score for star read(s) | : | -1.3 |
| Score for read counts | : | 0 |
| Score for mfe | : | 2 |
| Score for randfold | : | |
| Score for cons. seed | : | -0.6 |
| Total read count | : | 58 |
| Mature read count | : | 32 |
| Loop read count | : | 26 |
| Star read count | : | 0 |

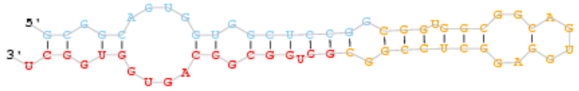

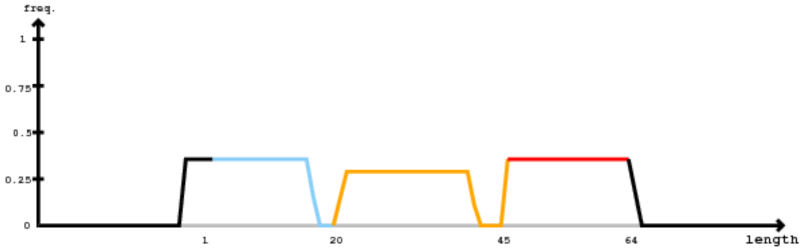


Star Mature

```
5'- gcggcaguggaggauccggugug gcggcagugguggcuccgg cgguggcggcaguggaggcuccggc gguggcggcagugguggcu ccggcgguagcggcaguggugg  -3'   exp
    .((.((.((..(...(((.(((.(.((.((.((.((.(.(((.(((.(((..(....)..)))))).))).).)).)).)).)).).))).)))...)..)).)).)).   reads   mm   sample
    ...................gguggcggcagugguggcu......................................................................   36   0   seq
    ...................gguggcggcagugguggcuc.....................................................................   28   0   seq
    ...........................................gguggcggcaguggaggcu..............................................   16   0   seq
    ...........................................gguggcggcaguggaggcuc.............................................   10   0   seq
    ...................................................................gguggcggcagugguggcu......................   36   0   seq
    ...................................................................gguggcggcagugguggcuc.....................   28   0   seq
```

novel_mir11

| | | |
|---|---|---|
| Provisional ID | : scaffold15_17748 | |
| Score total | : | 0.2 |
| Score for star read(s) | : | -1.3 |
| Score for read counts | : | 0 |
| Score for mfe | : | 2.1 |
| Score for randfold | : | |
| Score for cons. seed | : | -0.6 |
| Total read count | : | 158 |
| Mature read count | : | 116 |
| Loop read count | : | 42 |
| Star read count | : | 0 |

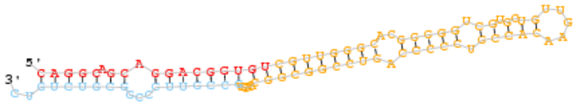


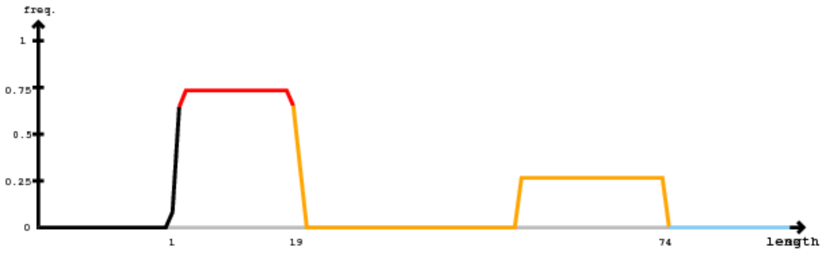


```
                   Mature                                                               Star
5'- acaucguuggcgucccaaucaggcagcaggacggugucguugggcacgggggucgugcuguugaacaccgucccccaguccggcggcaacgugccguucccggcgucugucа  -3'    exp
    .....(((((....)))))(((((.((.((((((((((((((((..(((((.((.(.((.....))))).))))).))))))))))).....)))))))...)))))))...   reads   mm   sample
    ..................Acaggcagcaggacggug............................................................................   13      1    seq
    ...................caggcagcaggacggugG...........................................................................   14      1    seq
    ...................cagAcagcaggacggugu...........................................................................   59      1    seq
    ...................caggcagcaggacggugGc..........................................................................   16      1    seq
    ....................aggcagcaggacggugGc..........................................................................   14      1    seq
    ......................................................................ucccccaguccggcggcaacgu....................   32      0    seq
    ......................................................................ucccccaguccggcggcGacgu....................   10      1    seq
```

novel_mir12

| | | |
|---|---|---|
| Provisional ID | : | scaffold205_28917 |
| Score total | : | 59106.5 |
| Score for star read(s) | : | 3.9 |
| Score for read counts | : | 59101.1 |
| Score for mfe | : | 2.1 |
| Score for randfold | : | |
| Score for cons. seed | : | -0.6 |
| Total read count | : | 115936 |
| Mature read count | : | 107531 |
| Loop read count | : | 0 |
| Star read count | : | 8405 |

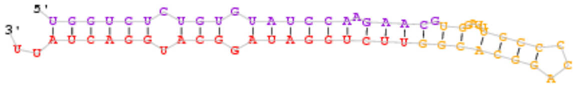


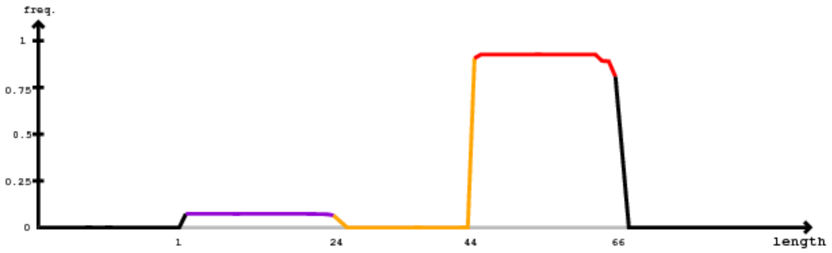


**Star** **Mature**

```
5'- gucccaggcacgguucuggauggucucuguguauccaagaacguggauuugccccaggcacgguucuggauaggcauggacuauucaguacagugcaugagcaagguucugga -3'   obs
    gucccaggcacgguucuggauggucucuguguauccaagaacguggauuugccccaggcacgguucuggauaggcauggacuauucaguacagugcaugagcaagguucugga        exp
    ...((..((((.((.(((((((((((.(((.((((((.((((.((....((((...)))))))))))))))).))).))))))))))).)).))))..((((...)))).)).   reads   mm   sample
    ......ggcacgguucuggauAgucucu.....................................................................................   11      1    seq
    .........acgguucuggauAgucucu.....................................................................................   10      1    seq
    ....................uggucucuguguauccaag..........................................................................   17      0    seq
    ....................uggucucuguguauccaaga.........................................................................   121     0    seq
    ....................uggucucuguguauccaagaa........................................................................   18      0    seq
    ....................uAgucucuguguauccaagaac.......................................................................   73      1    seq
    ....................uggucucuguguauccaagaac.......................................................................   509     0    seq
    ....................uggucucugCguauccaagaac.......................................................................   63      1    seq
    ....................uggucucuguguauccaagaaA.......................................................................   87      1    seq
    ....................uggucucuguguauccaagaaU.......................................................................   45      1    seq
    ....................uggCcucuguguauccaagaacg......................................................................   12      1    seq
    ....................uggucucugCguauccaagaacg......................................................................   332     1    seq
    ....................uAgucucuguguauccaagaacg......................................................................   261     1    seq
    ....................uggucucuguguauccaagaacU......................................................................   55      1    seq
    ....................uggucucuguguauccGagaacg......................................................................   10      1    seq
    ....................uggucucuguguauccaagaacg......................................................................   4210    0    seq
    ....................uggucucuguguauccaagaacC......................................................................   12      1    seq
    ....................uggucucugCguauccaagaacgu.....................................................................   22      1    seq
    ....................uggucucuguguauccaagaacgG.....................................................................   37      1    seq
    ....................uAgucucuguguauccaagaacgu.....................................................................   67      1    seq
    ....................uggucucuguguauccaagaacgA.....................................................................   348     1    seq
    ....................uggucucuguguauccaagaacgC.....................................................................   167     1    seq
    ....................uggucucuguguauccaagaacgu.....................................................................   1939    0    seq
    .......................................................aggcacgguucuggauag........................................   13      0    seq
    ..............................................................Cuucuggauaggcauggacuauu............................   45      1    seq
    ...............................................................uucuggauaggcauggacG...............................   18      1    seq
    ...............................................................uucuggauGggcauggacu...............................   19      1    seq
    ...............................................................uucuggauaggcauggacu...............................   3476    0    seq
    ...............................................................uucuggauaggcauggacA...............................   143     1    seq
    ...............................................................Cucuggauaggcauggacu...............................   10      1    seq
    ...............................................................uucuggauaggcauggacC...............................   363     1    seq
    ...............................................................uucuggauaggcauggacua..............................   234     0    seq
    ...............................................................uucuggGuaggcauggacuau.............................   30      1    seq
```

novel_mir13

| | | |
|---|---|---|
| Provisional ID | : | scaffold123_10915 |
| Score total | : | 805.6 |
| Score for star read(s) | : | 3.9 |
| Score for read counts | : | 801.1 |
| Score for mfe | : | 1.2 |
| Score for randfold | : | |
| Score for cons. seed | : | -0.6 |
| Total read count | : | 1583 |
| Mature read count | : | 1080 |
| Loop read count | : | 0 |
| Star read count | : | 503 |

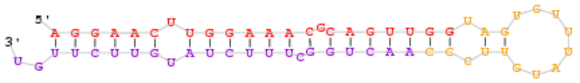


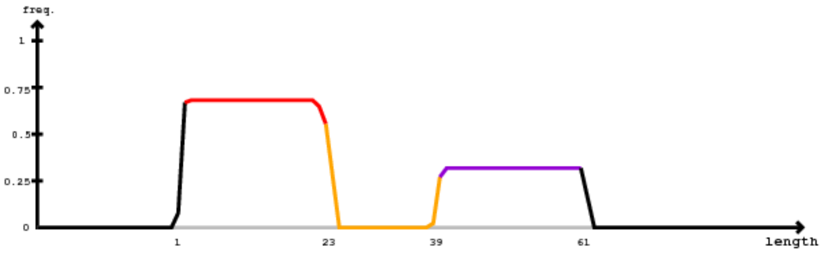


```
                    Mature                          Star
5'- aacucugcucauuugucaauaggaacuuggaaacgcaguugguaguguuuauguucccaacuggcuuucuauguucuuguuggaaugggugacuugcaucgcuguuaaaguu  -3'  obs
    aacucugcucauuugucaauaggaacuuggaaacgcaguugguaguguuuauguucccaacuggcuuucuauguucuuguuggaaugggugacuugcaucgcuguuaaaguu       exp
    ......((((((((..((((((((((.(((((((.(((((((.((........)).)))))))).)))))).))))))))))))))))))((((((((....)))..)))))   reads  mm  sample
    ...................uaggaacuuggaaacgcaguu........................................................................   17     0   seq
    ...................uaggaacuuggaaacgcaguug.......................................................................   59     0   seq
    ...................uaggaacuuggaaaUgcaguug.......................................................................   10     1   seq
    ...................uaggaacuuggaaacgcaguugA......................................................................   21     1   seq
    ...................uaggaacuuggaaacgcaguugU......................................................................   15     1   seq
    ....................aggaacuuggaaacgcaguu........................................................................   36     0   seq
    ....................aggaacuuggaaacgcaguug.......................................................................   81     0   seq
    ....................aggaacuuggaaacgcaguugg......................................................................   423    0   seq
    ....................aggaacuuggaaacgcaguuggC.....................................................................   10     1   seq
    ....................aggaacuuggaaacgcaguuggA.....................................................................   72     1   seq
    ....................aggaacuuggaaacgcaguuggu.....................................................................   316    0   seq
    .....................ggaacuuggaaacgcaguuggu.....................................................................   20     0   seq
    .........................................................caacuggcuuucuauguucuugu................................   35     0   seq
    ..........................................................aacuggcuuucuauguucuugA................................   28     1   seq
    ..........................................................aacuggcuuucuauguucuugu................................   355    0   seq
    ..........................................................aacuggcuuucuauguucuugC................................   10     1   seq
    ...........................................................acuggcuuucuauguucuugA................................   17     1   seq
    ...........................................................acuggcuuucuauguucuugu................................   58     0   seq
```

novel_mir14

| | | |
|---|---|---|
| Provisional ID | : | scaffold20_22912 |
| Score total | : | 3086.3 |
| Score for star read(s) | : | 3.9 |
| Score for read counts | : | 3081 |
| Score for mfe | : | 2 |
| Score for randfold | : | |
| Score for cons. seed | : | -0.6 |
| Total read count | : | 6055 |
| Mature read count | : | 6028 |
| Loop read count | : | 0 |
| Star read count | : | 27 |

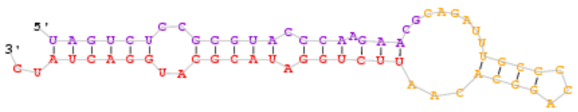


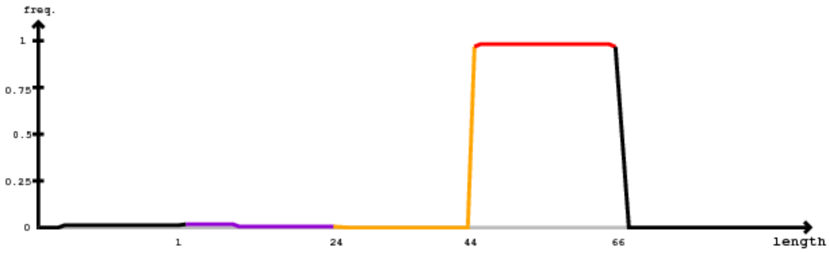


```
                  Star                                     Mature
5'- ggaucaagcacgguuguggauagucuccgcguacccaagaacgcagauuugccccaggcacaauucuggauacgcauggacuauccaaacccgugccugagcaagguucugga   -3'   obs
    ggaucaagcacgguuguggauagucuccgcguacccaagaacgcagauuugccccaggcacaauucuggauacgcauggacuauccaaacccgugccugagcaagguucugga         exp
    ...(((.((((((...((((((((((..(((((.(((.(((........((((...))))...)))))).)))))..))))))))))...)))))).))).............     reads    mm    sample
    ..aucaagcacgguuCuggauagucucc.....................................................................................     77       1     seq
    ....................uagucuccgcguacccaagaacg......................................................................     27       0     seq
    ...............................................................uucuggauaUgcauggacuau.............................     86       1     seq
    ...............................................................uucuggauaGgcauggacuauc............................     5843     1     seq
    ...............................................................uucuggauaUgcauggacuauc............................     14       1     seq
    ................................................................ucuggauacgUauggacuau.............................     11       1     seq
    ................................................................ucuggauaGgcauggacuauc............................     74       1     seq
```

novel_mir15

| | | |
|---|---|---|
| Provisional ID | : | scaffold26_665 |
| Score total | : | 390.1 |
| Score for star read(s) | : | 3.9 |
| Score for read counts | : | 384 |
| Score for mfe | : | 2.8 |
| Score for randfold | : | |
| Score for cons. seed | : | -0.6 |
| Total read count | : | 765 |
| Mature read count | : | 381 |
| Loop read count | : | 0 |
| Star read count | : | 384 |

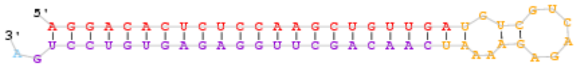


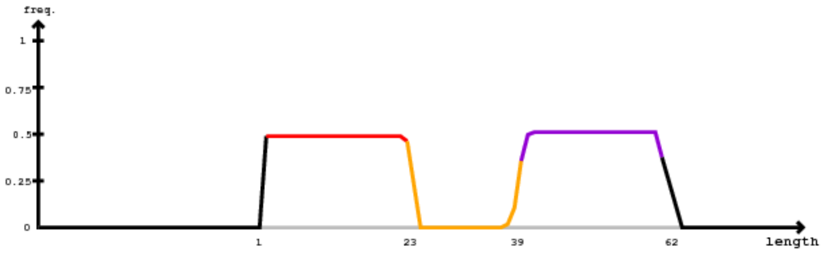


Mature Star

```
5'- uuuaaugggacaagcgcaaugcaacaucacucaggacacucuccaagcuguugaugucgucagagaaaaucaacagcuuggagaguguccugagugauguugcuuuucaaag   -3'   obs
    uuuaaugggacaagcgcaaugcaacaucacucaggacacucuccaagcuguugaugucgucagagaaaaucaacagcuuggagaguguccugagugauguugcuuuucaaag         exp
    .....((.(.....).))..((((((((((((((((((((((((((((((((((.((......))..))))))))))))))))))))))))))))))))))).........   reads   mm   sample
    ................................Gggacacucuccaagcuguug...........................................................   21      1    seq
    ................................Gggacacucuccaagcuguuga..........................................................   255     1    seq
    ................................aggacacucuccaagcuguuga..........................................................   105     0    seq
    ....................................................................aucaacGgcuuggagaguguccu.....................   14      1    seq
    .....................................................................ucaacGgcuuggagaguguccu.....................   53      1    seq
    .....................................................................ucaacagcuuggagaguguccu.....................   15      0    seq
    ......................................................................caacGgcuuggagaguguccu.....................   24      1    seq
    ......................................................................caacGgcuuggagaguguccug....................   45      1    seq
    ......................................................................caacagcuuggagaguguccug....................   87      0    seq
    ......................................................................caacagcuuggagaguguccuga...................   21      0    seq
    ......................................................................caacagcuuggagaguguccugU...................   19      1    seq
    .......................................................................aacagcuuggagaguguccug....................   11      0    seq
    .......................................................................aacagcuuggagaguguccugU...................   14      1    seq
    .......................................................................aacagcuuggagaguguccuga...................   84      0    seq
    ........................................................................acagcuuggagaguguccuga...................   11      0    seq
```

novel_mir16

| | | |
|---|---|---|
| Provisional ID | : | scaffold128_524 |
| Score total | : | 300.8 |
| Score for star read(s) | : | 3.9 |
| Score for read counts | : | 295.3 |
| Score for mfe | : | 2.2 |
| Score for randfold | : | |
| Score for cons. seed | : | -0.6 |
| Total read count | : | 591 |
| Mature read count | : | 316 |
| Loop read count | : | 0 |
| Star read count | : | 275 |

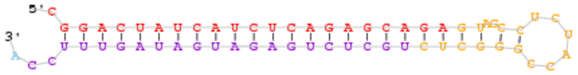


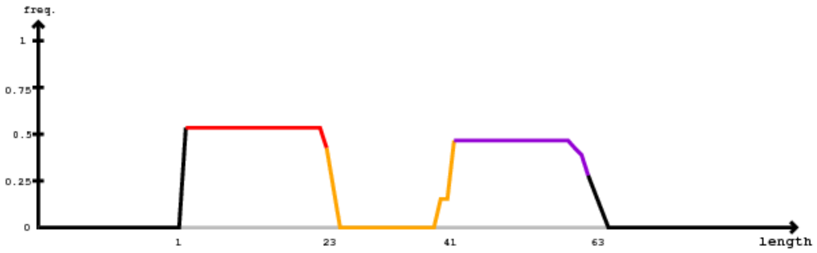


**Mature** **Star**

```
5'- guggcaguauguacucuaugcggacuaucaucucagagcagaguagccucuacgggcucugcucugagaugauaguuuccaguaguaacuaucaucucagagcagaguag  -3'   obs
    guggcaguauguacucuaugcggacuaucaucucagagcagaguagccucuacgggcucugcucugagaugauaguuuccaguaguaacuaucaucucagagcagaguag         exp
    (((..(((...((((...((..((((((((((((((((((((((..((......))))))))))))))))))))))))..))..)))))))..)))(((......)))...   reads   mm   sample
    ....................cggacuaucaucucagagcag.....................................................................   65      0    seq
    ....................cggacuaucaucucagagcaga....................................................................   251     0    seq
    ........................................................ucugcucugagaugauaguu..................................   24      0    seq
    ........................................................ucugcucugagaugauaguuuc................................   16      0    seq
    ........................................................ucugcucugagaugauaguuAc................................   50      1    seq
    ..........................................................ugcucugagaugauaguuu.................................   21      0    seq
    ..........................................................ugcucugagaugauaguuucc...............................   131     0    seq
    ..........................................................ugcucugagaugauaguuucca..............................   19      0    seq
    ..........................................................ugcucugagaugauaguuuccaU.............................   14      1    seq
```

novel_mir17

| | | |
|---|---|---|
| Provisional ID | : | scaffold21_29550 |
| Score total | : | 296.4 |
| Score for star read(s) | : | 3.9 |
| Score for read counts | : | 291.2 |
| Score for mfe | : | 1.9 |
| Score for randfold | : | |
| Score for cons. seed | : | -0.6 |
| Total read count | : | 583 |
| Mature read count | : | 464 |
| Loop read count | : | 0 |
| Star read count | : | 119 |

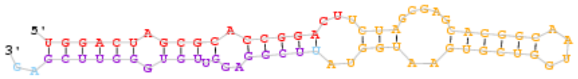

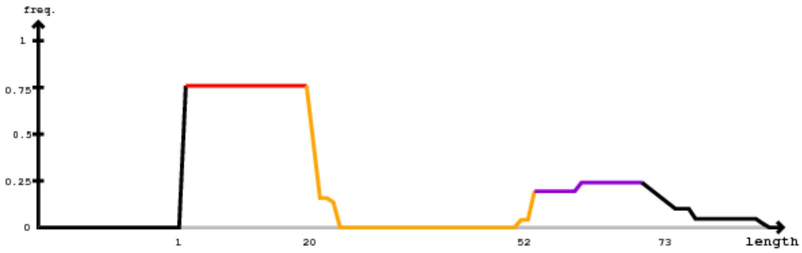


```
                         Mature                                           Star
5'- ggcgaguccucguggcgcaauggacuagcgcaccggacuucuagcgagcacggcaauggucgugaaugguauucggagguuguggguucgagucccaccgaggacggcu  -3'   obs
    ggcgaguccucguggcgcaauggacuagcgcaccggacuucuagcgagcacggcaauggucgugaaugguauucggagguuguggguucgagucccaccgaggacggcu         exp
    (((..((((((((((.((..((((((.((((.(((((...(((.....((((((....))))))..)))..)))))..).))).)))))).)).))).))))))).)))   reads   mm   sample
    ....................uggacuagcgcaccggacu......................................................................   276     0    seq
    ....................uggacuagcgcaccggacuu.....................................................................   91      0    seq
    ....................uggacuagcgcaccggacuucu...................................................................   15      0    seq
    ....................uggacuagcgcaccggacuucua..................................................................   82      0    seq
    ......................................................................auucggagguuguggguuc....................   15      0    seq
    ......................................................................auucggagguuguggguucg...................   10      0    seq
    ........................................................................ucggagguuguggguucg...................   61      0    seq
    ........................................................................ucggagguuguggguucgUguccc.............   22      1    seq
    ........................................................................ucggagguuguggguucgGguccc.............   11      1    seq
    ...............................................................................uuguggguucgagucccaccgaggacg...   15      0    seq
    ...............................................................................uuguggguucgagucccaccgaggacgC..   13      1    seq
```

novel_mir18

| | | |
|---|---|---|
| Provisional ID | : | scaffold19_20729 |
| Score total | : | 170.4 |
| Score for star read(s) | : | 3.9 |
| Score for read counts | : | 164.3 |
| Score for mfe | : | 2.8 |
| Score for randfold | : | |
| Score for cons. seed | : | -0.6 |
| Total read count | : | 334 |
| Mature read count | : | 310 |
| Loop read count | : | 0 |
| Star read count | : | 24 |

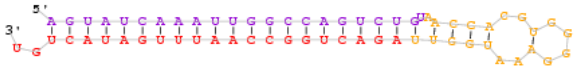

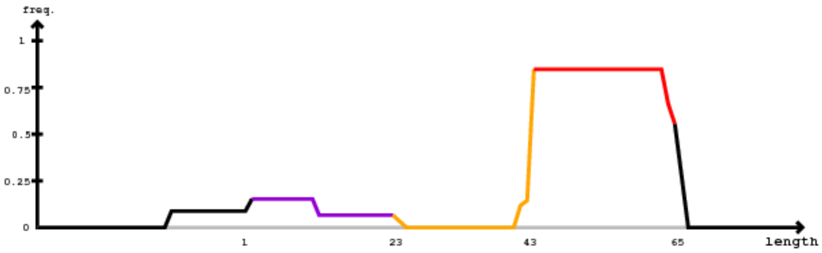


```
                                Star                                     Mature
5'- aagcagaucuuuuggugaugggggguaagacaguaucaaauuggccagucuguaaccacgugggggaaaugguuagacuggccaauuugauacugucuuacccacaccaaaaga   -3'   obs
    aagcagaucuuuuggugaugggggguaagacaguaucaaauuggccagucuguaaccacgugggggaaaugguuagacuggccaauuugauacugucuuacccacaccaaaaga         exp
    .......(((((((((....(((((((((((((((((((((((((((((..((((..(....)..)))))))))))))))))))))))))))))))))))).))))))))))   reads   mm   sample
    ..................uggggguaagacaguaucaaau.............................................................................   32      0    seq
    ..............................aguaucaaauuggccagucugu.................................................................   24      0    seq
    ..........................................................................uuagacuggccaauuugauacu...................   43      0    seq
    ...........................................................................uagacuggccaauuugauacu...................   10      0    seq
    ............................................................................agacuggccaauuugauacu...................   14      0    seq
    ............................................................................agacuggccaauuugauacug..................   40      0    seq
    ............................................................................agacuggccaauuugauacugu.................   203     0    seq
```

novel_mir19

| | | |
|---|---|---|
| Provisional ID | : | scaffold40_28810 |
| Score total | : | 85.6 |
| Score for star read(s) | : | 3.9 |
| Score for read counts | : | 80.7 |
| Score for mfe | : | 1.7 |
| Score for randfold | : | |
| Score for cons. seed | : | -0.6 |
| Total read count | : | 170 |
| Mature read count | : | 112 |
| Loop read count | : | 0 |
| Star read count | : | 58 |

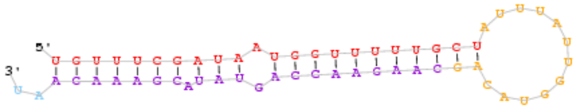


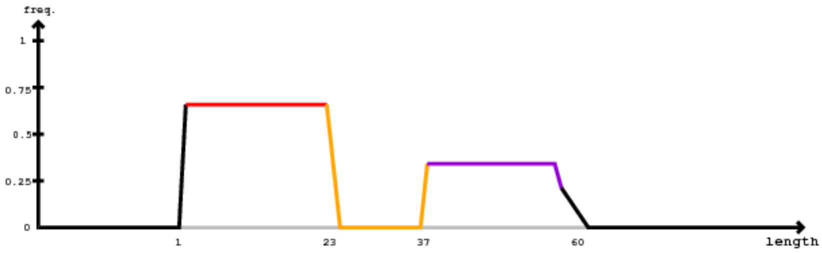


```
                  Mature                          Star
5'- uauauuuugguacgguugcuuguuucgauaaugguuuuugcuauuuauugguacagcaagaaccaguauacgaaacaaucaaccguuucccaucagcaacaucacauuuuac  -3'   obs
    uauauuuugguacgguugcuuguuucgauaaugguuuuugcuauuuauugguacagcaagaaccaguauacgaaacaaucaaccguuucccaucagcaacaucacauuuuac        exp
    .......(((.(((((((.(((((((((((.(((((((((((...........))))))))))).))).)))))))).)))))))...)))....................  reads  mm  sample
    ....................uguuucgauaaugguuuuugcu......................................................................  101    0   seq
    ....................uguuucgauaaugguuuuugcG......................................................................  11     1   seq
    ........................................................caagaaccaguauacgaaac....................................  22     0   seq
    ........................................................caagaaccaguauacgaaaca...................................  36     0   seq
```

novel_mir20

| | | |
|---|---|---|
| Provisional ID | : | scaffold127_35934 |
| Score total | : | 79.1 |
| Score for star read(s) | : | 3.9 |
| Score for read counts | : | 73 |
| Score for mfe | : | 2.8 |
| Score for randfold | : | |
| Score for cons. seed | : | -0.6 |
| Total read count | : | 155 |
| Mature read count | : | 85 |
| Loop read count | : | 0 |
| Star read count | : | 70 |

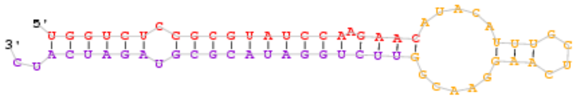


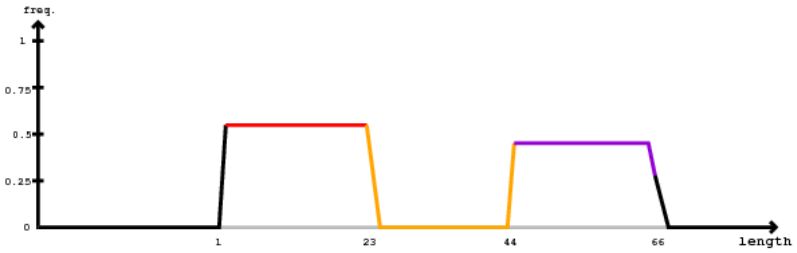


**Mature** **Star**

```
5'- ggguuaggaucaggcacgguucuggauggucuccgcguauccaagaacauacauuugcucaaggaacgguucuggauacgcguagaucauccagaaccgugccugauc   -3'   obs
    ggguuaggaucaggcacgguucuggauggucuccgcguauccaagaacauacauuugcucaaggaacgguucuggauacgcguagaucauccagaaccgugccugauc         exp
    .......((((((((((((((((((((((((.((((((((((.((((.....(((....))).....)))))))))))))).))))))))))))))))))))))))   reads   mm   sample
    ..........................uggucucUgcguauccaagaac............................................................   63      1    seq
    ..........................uggucucUgcguauccaagaaca...........................................................   22      1    seq
    .....................................................................uucuggauacgcguagauUau..................   27      1    seq
    .....................................................................uucuggauacgcguagauUauc.................   43      1    seq
```

novel_mir21

| | | |
|---|---|---|
| Provisional ID | : | scaffold106_22274 |
| Score total | : | 40.1 |
| Score for star read(s) | : | 3.9 |
| Score for read counts | : | 34.3 |
| Score for mfe | : | 2.5 |
| Score for randfold | : | |
| Score for cons. seed | : | -0.6 |
| Total read count | : | 79 |
| Mature read count | : | 68 |
| Loop read count | : | 0 |
| Star read count | : | 11 |

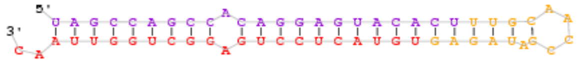


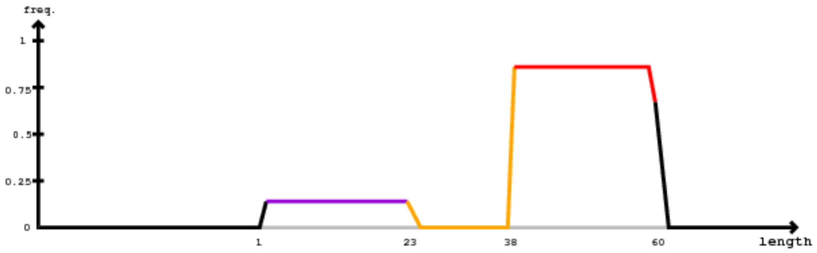


```
                                Star                                    Mature
5'- auaauaauuuauguugaauauaccauaggaguuagccagccacaggaguacacuuugcaaccgauagaguguacuccugaggcugguuaacuccucugguagaucauuccg  -3'   obs
    auaauaauuuauguugaauauaccauaggaguuagccagccacaggaguacacuuugcaaccgauagaguguacuccugaggcugguuaacuccucugguagaucauuccg         exp
    ..............(((...(((((.(((((((((((((((.((((((((((((((((....).))))))))))))))).))))))))))))))).)))))..))).....   reads   mm   sample
    ................................uagccagccacaggaguacacu.........................................................   11      0    seq
    .....................................................................uguacuccugaggcugguuaU.....................   15      1    seq
    .....................................................................uguacuccugaggcugguuaaU....................   40      1    seq
    .....................................................................uguacuccugaggcugguuaac....................   13      0    seq
```

novel_mir22

| | | |
|---|---|---|
| Provisional ID | : | scaffold12_40947 |
| Score total | : | 19.7 |
| Score for star read(s) | : | 3.9 |
| Score for read counts | : | 13.9 |
| Score for mfe | : | 2.5 |
| Score for randfold | : | |
| Score for cons. seed | : | -0.6 |
| Total read count | : | 39 |
| Mature read count | : | 25 |
| Loop read count | : | 0 |
| Star read count | : | 14 |

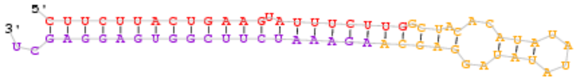


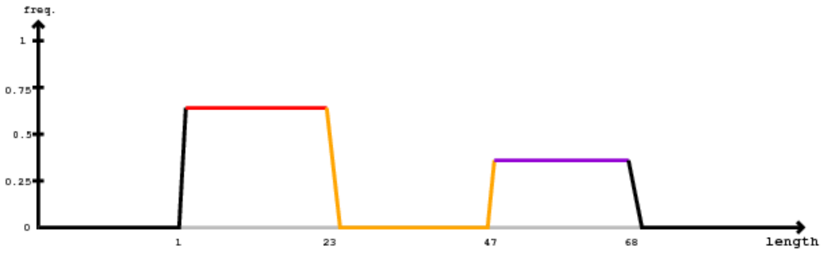


Mature Star

```
5'- aucagacaccuuccucagagcuucuuacugaaguauuucuuggcuacacauauauauauaggagcaagaaaucuucggugaggagcucugaggaagguagacugacaaguag  -3'  obs
    aucagacaccuuccucagagcuucuuacugaaguauuucuuggcuacacauauauauauaggagcaagaaaucuucggugaggagcucugaggaagguagacugacaaguag       exp
    .((((.(((((((((((((((((((((((((((.((((((((.((.(..(((....)))..))))))))))))))))))))))))))))))))))))).).)))).......  reads  mm  sample
    ....................cuucuuacugaaguauuucuug......................................................................  25     0   seq
    ..................................................................agaaGucuucggugaggagcu.........................  14     1   seq
```

novel_mir23
