# Supplementary figures and images for: Genome-Wide Identification and Transcriptomic Analysis of MicroRNAs Across Various Amphioxus Organs Using Deep Sequencing
Source: Front Genet. 2019 Sep 26;10:877. doi: 10.3389/fgene.2019.00877 (PMC6775235; doi:10.3389/fgene.2019.00877)

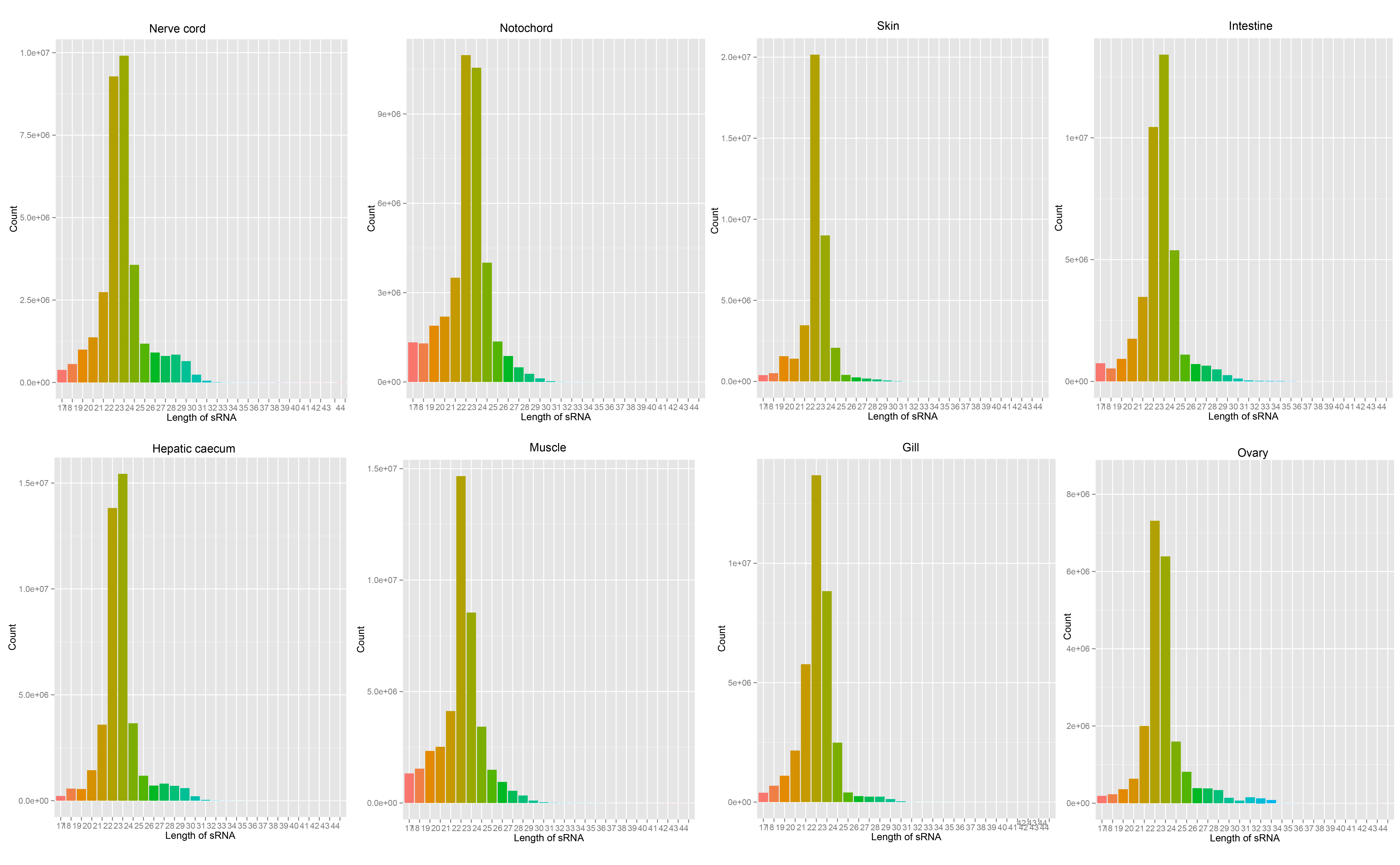

Supplement: Supplementary File 1 — Taqman probe information of randomly selected miRNAs used in qRT-PCR analysis. [file Presentation_1.zip › Supplementary File 3.JPEG]

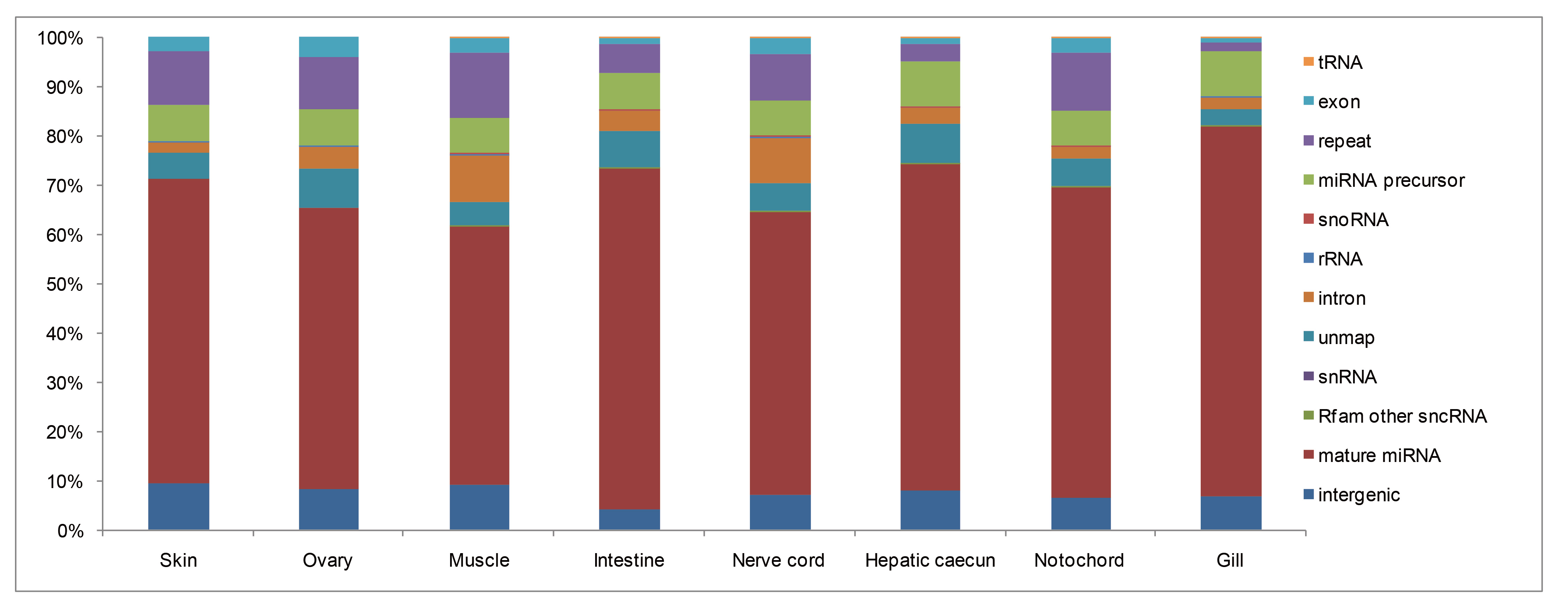

Supplement: Supplementary File 1 — Taqman probe information of randomly selected miRNAs used in qRT-PCR analysis. [file Presentation_1.zip › Supplementary File 4.JPEG]
